# Microstructure and properties evolution of Mg–2Y–0.6Nd–0.6Zr alloy rolled at room and liquid nitrogen temperature

**DOI:** 10.1038/s41598-021-99706-x

**Published:** 2021-10-22

**Authors:** Yun Tan, Wei Li, Aiwen Li, Xiaofang Shi

**Affiliations:** 1 College of Material and Metallurgy, Guizhou University, Guiyang, 550025 China; 2 AECC Guizhou Liyang Avitation Power Co., Ltd., Guiyang, 550014 China; 3 Guizhou Key Laboratory for Mechanical Behavior and Microstructure of Materials, Guizhou University, Guiyang, China; 4 National and Local Joint Engineering Laboratory for High-Performance Metal Structure Material and Advanced Manufacturing Technology, Guiyang, China

**Keywords:** Engineering, Materials science

## Abstract

The microstructure evolution, texture, mechanical behavior and twin deformation of the ECAPed Mg–2Y–0.6Nd–0.6Zr alloy at room and liquid nitrogen temperature were investigated by rolling samples. The ECAP processed material appeared the texture of 45° to the extrusion direction and its yield strength reached 93.6 MPa. The results showed that cryorolling encourages twinning in Mg–2Y–0.6Nd–0.6Zr alloy, enhancing the tensile strength and texture. Activation of {10–12} twinning during rolling was found to be more pronounced in the cryorolled samples than in the cold rolled samples owing to a lower temperature. As a result, the cryorolled samples had more twins than and cold rolled ones, the proportion of twin areas of room temperature rolling and ultra-low temperature rolling were: 2.45% and 4.23%.

## Introduction

Magnesium (Mg) alloys are currently the lightest commercial metal structural material. At the same time, it has high specific strength, good damping and vibration reduction, good thermal conductivity and good electronic shielding effects^1–4^. Therefore, Mg alloys have attracted increasing attention in aerospace, pharmaceutical, chemical and automotive fields in recent years^5–7^. However, the limited number of available slipping systems at room temperatures due to its hexagonal close-packed (HCP) structure leads to poor deformability^8–10^. Therefore, it is of great significance to study the plastic deformation mechanism and mechanical properties of magnesium alloys at low temperature. Still further work is needed in this area since, for example, the evolution of texture and twinning in Mg alloys materials during cryorolling is still not thoroughly understood.

In recent years, some researchers have also conducted a series of studies on the plastic deformation of Mg and Mg alloys at room temperature. Volkov et al.^11^ used cold hydrostatic extrusion and low-temperature annealing to improve the mechanical properties of pure magnesium. The results of the study found that the pure magnesium was deformed at a lower temperature to produce ultrafine-grained pure magnesium with 3 μm and high plasticity. Antonova et al.^12^ performed severe plastic deformation extrusion on three pure magnesium samples with different columnar structure orientations at room temperature. The study showed that activation of twins during deformation changed the orientation of the original grains, which was more conducive to sliding. In most regions, sub-structures such as 100 nm cells are found. Under the condition of high load value, it may have provided the necessary stresses for activation of non-basal plane slip systems with high Schmid factor, and the grain size is significantly refined, reaching more than two orders of value to the initial size. Zhu et al.^13^ showed that dynamic twin boundaries and stacking faults are conducive to the formation of low-angle grain boundaries, which can be transformed into high-angle grain boundaries and form ultra-fine dynamic recrystallized grains. Wang et al.^14^ showed that for the grains of about 6–22 μm, the dislocation slip is mainly dominated during the deformation process. However, for the grains of about 22–41 μm, the twins are mainly dominated by the deformation process.

Mg alloys exhibit different deformation mechanisms and mechanical behaviors at different temperatures. There is no doubt that temperature is an important factor that affecting the deformation mechanism of magnesium alloys with different properties. In addition, many studies have reported the deformation temperatures of magnesium alloys are at room temperature and above, but few studies have reported mechanical behavior and deformation mechanisms of a rolled Mg alloys at low temperatures, especially ultra-low temperatures (liquid nitrogen temperature). Therefore, the present study systematically investigates the evolution and mechanical properties of the fine-grained Mg–2Y–0.6Nd–0.6Zr alloy under low temperature rolling deformation, such as microstructure, twinning, texture, and explores the plastic deformation mechanism and twinning behavior of Mg alloy at room temperature and ultra-low temperature.

## Materials and methods

**Figure 1:**
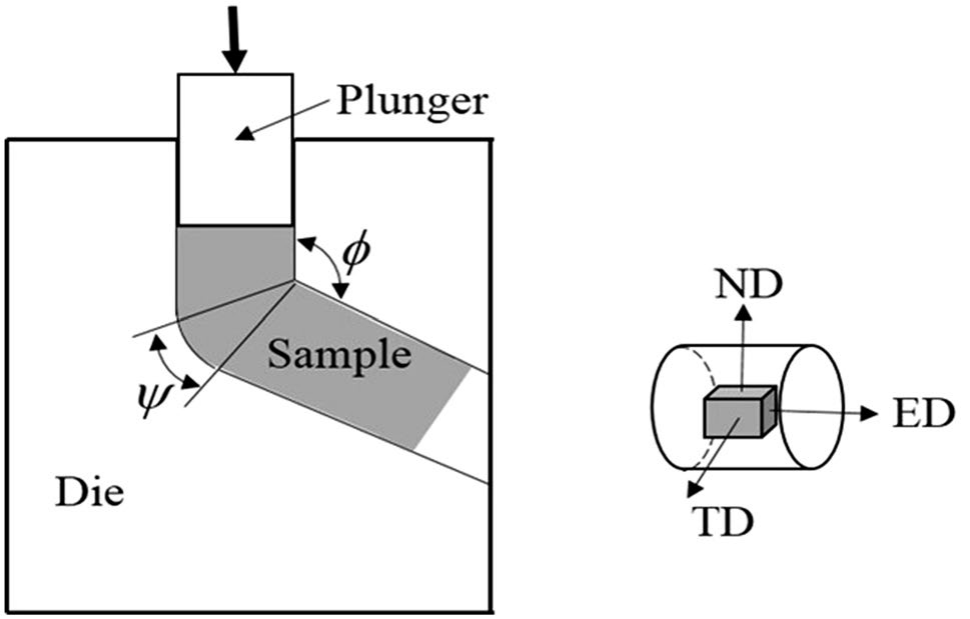
ECAP mold structure diagram.

The as-received material used in this study was as-cast Mg–2Y–0.6Nd–0.6Zr alloy, which was smelted by low-pressure casting and introduced with SF6 and CO_2_ gas protection. Rod-shaped magnesium alloys with a size of ϕ12 mm × 80 mm were cut out of the initial material for ECAP. The samples were homogenized at 450 °C for 6 h and then cooled in air. After that, the Mg–2Y–0.6Nd–0.6Zr alloy bar was placed into an ECAP die and pressed at 400 °C for four passes by BC route (90° anticlockwise rotation of the samples after each ECAP pass) under a compressive speed of 0.4 mm/s. The ECAP die structure used is shown in Fig. 1, where φ = 120°, Ψ = 30°. Finally, the ECAPed Mg alloys were rolled in two passes from 12 to 10 mm in diameter at ultra-low temperature (liquid nitrogen immersion) and room temperature, respectively. The total deformation of rolling was 16.7%. In the ultra-low temperature rolling process, the samples were immersed in liquid nitrogen for 10–15 min before each rolling.

The microstructures and textures of the processed specimens were observed on the ND–ED(RD) plane using optical microscopy (OM), electron backscatter diffraction (EBSD). ND and ED (RD) represent the normal direction and extrusion direction (rolling direction) of the extruded sample. Additionally, for OM the grain structure was revealed by subsequent etching using a solution of ethanol (14 ml), water (2 ml), glacial acetic acid (4 ml) and picric acid (0.82 g). The grain size was measured and calculated using Image-Pro Plus image analysis software. Electron back-scattered diffraction (EBSD) analysis were performed on a S-3400N scanning electron microscope using Channel 5and Mtex software to acquire and analysis data. The EBSD sample was prepared by ion etching. The tensile samples were machined along the pressing direction, tensile testing was carried out at room temperature (RT) at an initial strain rate of 3 × 10^−3^ s^−1^ using an electronic universal Instron 8501 testing machine.

## Experimental results and discussion

### Microstructure

**Figure 2:**
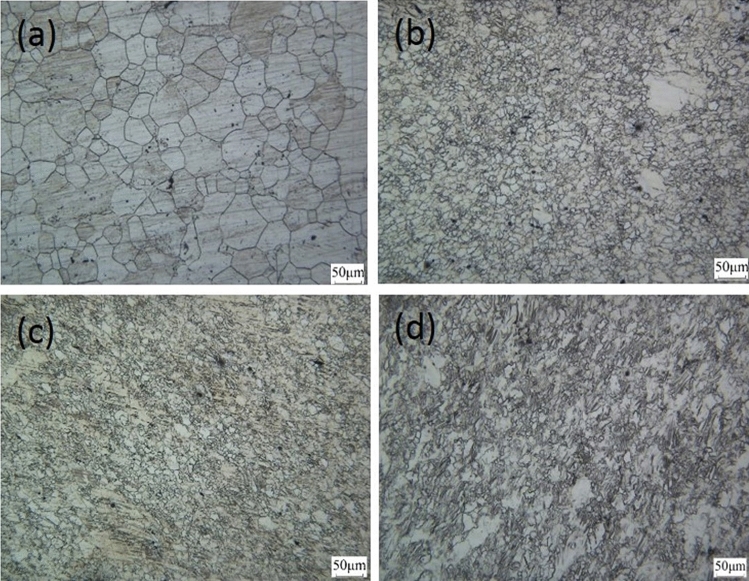
Microstructure of magnesium alloy in different states. (**a**) Homogenization, (**b**) ECAP4passes, (**c**) ECAP4Passes + room temperature rolling, (**d**) ECAP4Passes + liquid nitrogen rolling.

Figure 2 shows the microstructure of Mg–2Y–0.6Nd–0.6Zr alloy by using the OM techniques. The average grain size for as-annealed Mg alloy was calculated to be 52 μm. After 4 passes equal channel angular pressing, as shown in Fig. 2b, the effect of shear stress and dynamic recrystallization make the grains significantly refined, Consequently, the average grain size decreases from 52 to 10 μm. After rolling at room temperature and rolling with liquid nitrogen, different degrees of twinning appeared, and the grains were also elongated, but the change in grain size is not obvious, about 10um. Comparing Fig. 2c with Fig. 2d, it can be found that the cryorolled samples had more twins than and cold rolled ones, but there is no obvious twins in the samples rolled at room temperature. In short, when the size of the magnesium alloy is 10 μm, the lower the deformation temperature, the easier the twins appear.

**Figure 3:**
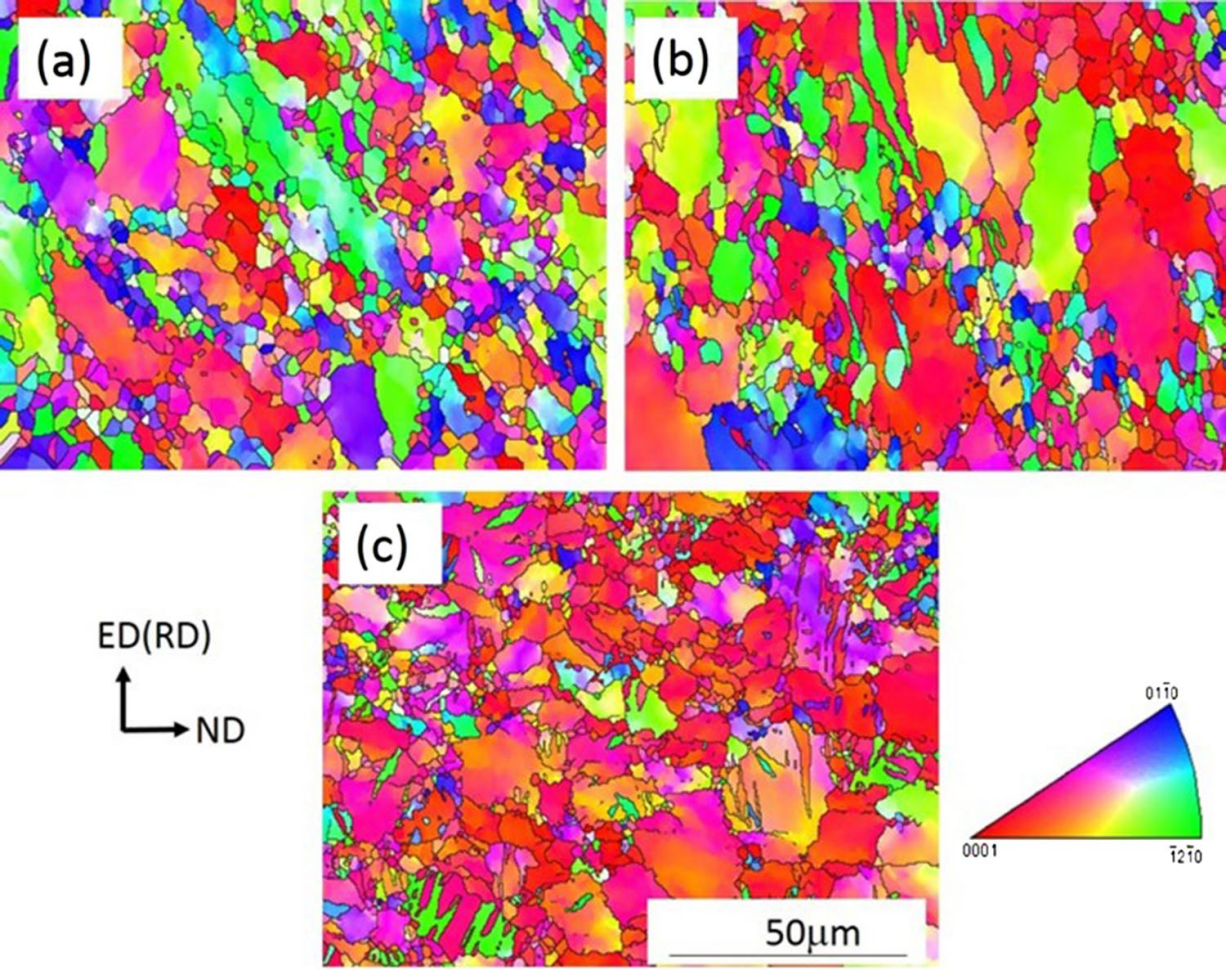
EBSD grain orientation diagram of Mg–2Y–0.6Nd–0.6Zr alloy in different states. (**a**) ECAP4passes, (**b**) ECAP4Passes + rolling, (**c**) ECAP4Passes + liquid nitrogen.

Figure 3 shows the EBSD inverse pole figure map of the Mg–2Y–0.6Nd–0.6Zr alloy in different states, the observation surface is ED (RD)–ND. As shown in Fig. 3a, the large grains in the original homogenized structure are directly broken into tens of substructures, and there are many sub-structures inside the large grains after 4 ECAP passes processing, and the accumulated strain and deformation storage energy increase, the effect of dynamic recrystallization is obvious, so that more fine dynamic recrystallized grains are formed and grown, and the grain size is refined to 10 μm^15^. However, for rolling at different temperatures, the grain size of the sample does not change significantly, and twins appear inside the grains, which are mirror-like, as shown in Fig. 3b and c, it was observed that the number of twins appeared in ultra-low temperature rolling compared to room temperature rolling.

### Texture change of Mg–2Y–0.6Nd–0.6Z alloy at low temperature

**Figure 4:**
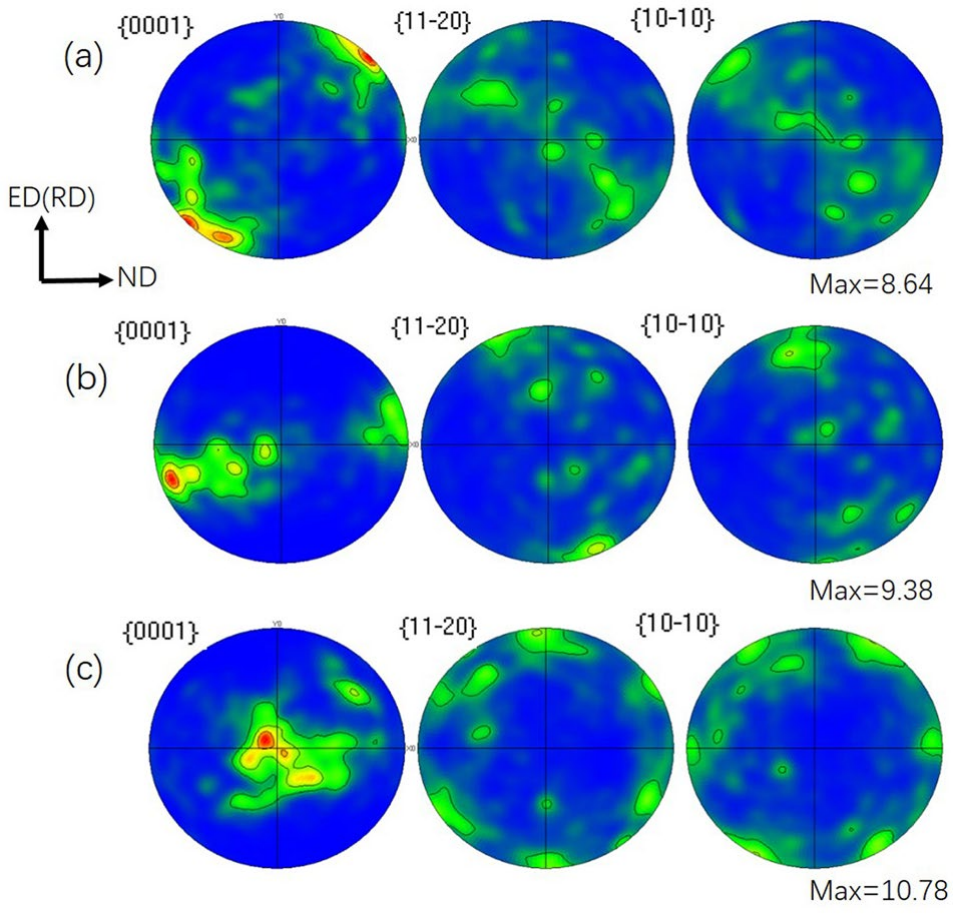
EBSD microscopic pole figure of the sample in different deformation states of Mg–2Y–0.6Nd–0.6Zr alloy. (**a**) ECAP4passes, (**b**) ECAP4Passes + rolling, (**c**) ECAP4Passes + liquid nitrogen.

Figure 4 shows the pole figures of the Mg–2Y–0.6Nd–0.6Zr alloy. It can be clearly seen that after ECAP for 4 passes using route BC, a texture with an angle of about 45° to the extrusion direction (ED) appears on the (0001) pole figure and exhibits a strong basal texture of 8.64. In contrast, the as-ECAPed textures of Mg–2Y–0.6Nd–0.6Zr alloy in (0001), (10$\overline{1}$0) and (10$\overline{1}$1) planes are slightly weaker than the rolled texture. With decreasing the deformation temperature, the strongest pole was gradually pulled back to the center, and the typical basal surface wire texture appeared, and the pole density increasing from 8.64 to 10.78. Thus, it can be concluded that ultra-low temperature rolling treatment can increase the texture^16^.

### Deformation twinning of magnesium alloy at low temperature

**Figure 5:**
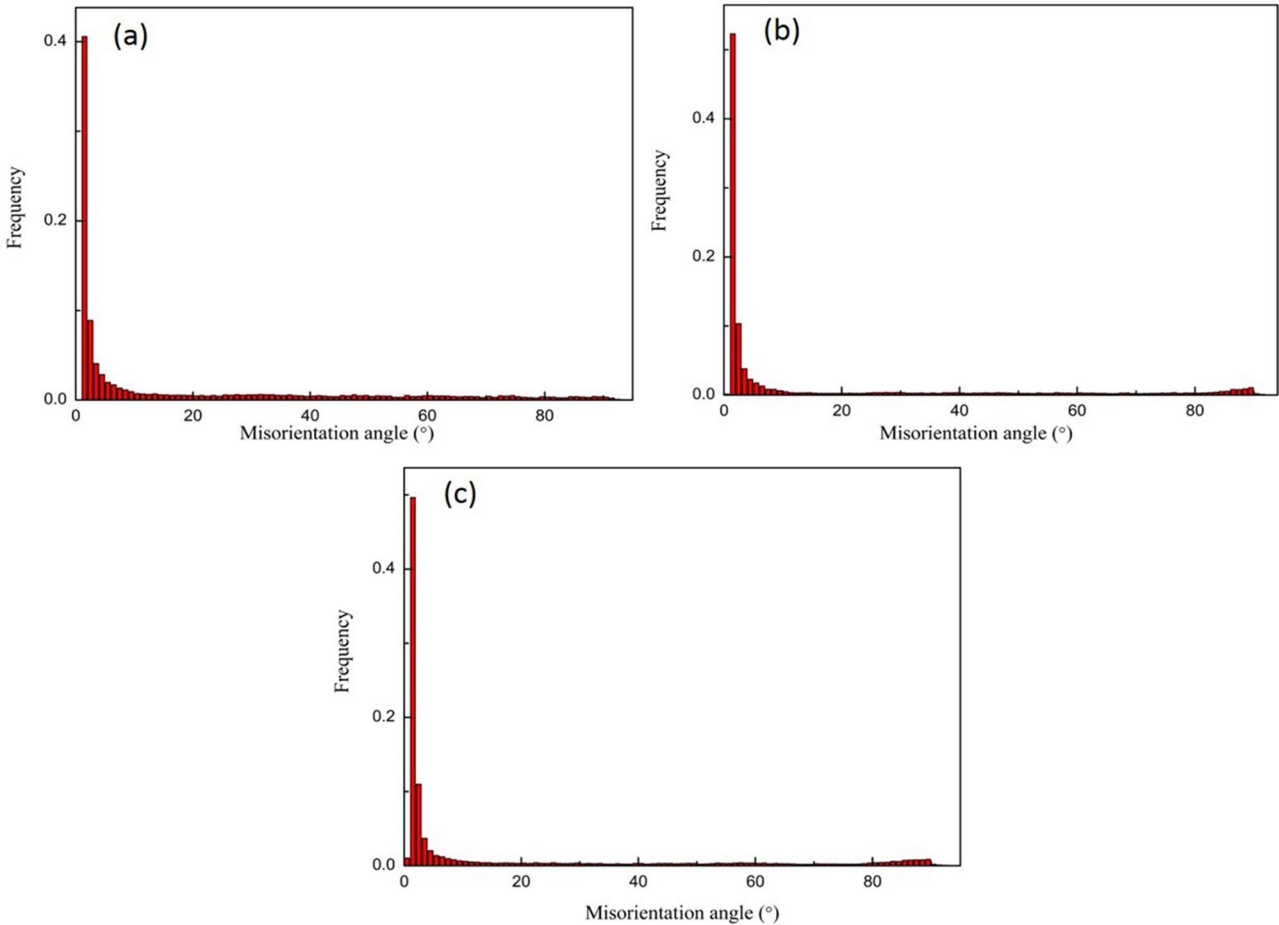
Distribution of orientation difference of Mg–2Y–0.6Nd–0.6Zr alloy after ECAP at different rolling temperatures. (**a**) ECAP4passes, (**b**) ECAP4Passes + rolling, (**c**) ECAP4Passes + liquid nitrogen.

Figure 5 shows the distribution of orientation difference of Mg–2Y–0.6Nd–0.6Zr alloy at different rolling temperatures after ECAP. It can be seen that the proportion of low angle grain boundaries (LAGBs) is 63.4% after 4 passes of equal channel angular pressing. After rolling, the proportion of high angle grain boundaries (HAGBs) decreases, and the proportion of LAGBs (< 10°) increase to about 72%. Of course, it can also be seen from the figure that the peak value at 86° increases obviously, which may also be due to the appearance of 86° tensile twin.

**Figure 6:**
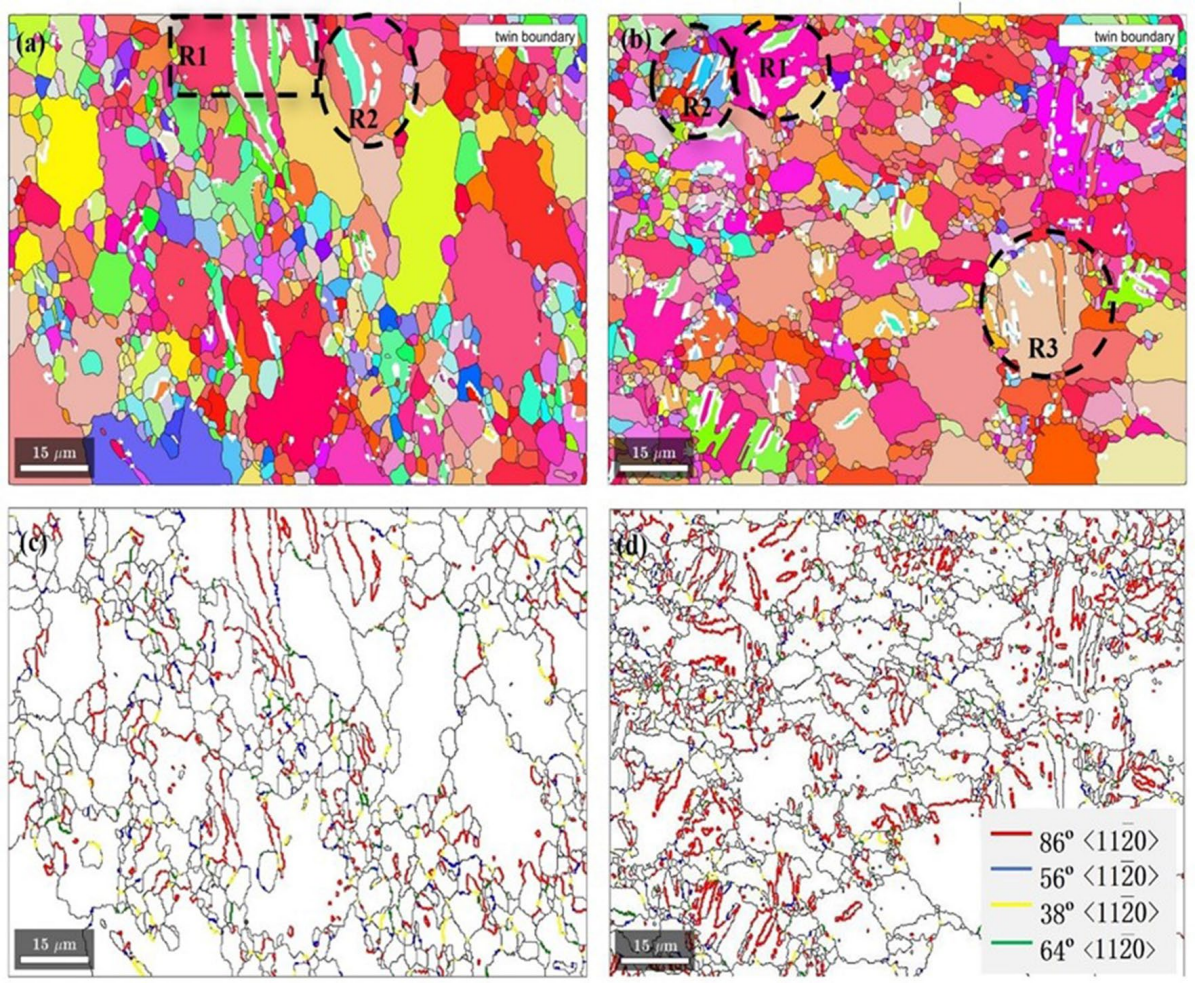
Twinning distribution of Mg–2Y–0.6Nd–0.6Zr alloy after ECAP under different rolling temperatures. (**a**), (**c**) ECAP4Passes + room temperature, (**b**), (**d**) ECAP4Passes + ultra-low temperature.

Figure 6 shows the twin distribution diagram of Mg–2Y–0.6Nd–0.6Zr alloy at different rolling temperatures after ECAP, in which the white grain boundary in Fig. 6a and b represents the twin boundary. The twin ratio of Mg–2Y–0.6Nd–0.6Zr alloy after statistically rolled at room temperature is 2.45%, and the twin ratio of ultra-low temperature rolling is 4.23%. Figure 6a and c are schematic diagrams of twins of the samples after rolling at room temperature. Some 86° <11$\overline{2}$0> twins and a few 56° <11$\overline{2}$0> twins and 38° <11$\overline{2}$0> twins can be observed during rolling at room temperature, while 64° <11$\overline{2}$0> twins are almost absent. It is also found that 86° <11$\overline{2}$0> twins basically exist in relatively large grains when rolled at room temperature, while there are few twins in small grains. The microstructure of the samples rolled at room temperature is not very uniform, and the large grains are stretched. Figure 6c shows the rolling deformation treatment at ultra-low temperature. A large number of twins are observed in the figure. Compared with the samples deformed at room temperature and high temperature, 86° <11$\overline{2}$0> tensile twins appear more frequently. With the decrease of deformation temperature, the basal slip is blocked. At this time, twins play a vital role and are more likely to occur, and the crystal rotation also adds deformation to change the crystal orientation. At room temperature, the CRSS required to start the non-basal slip system of magnesium and its alloys is about 100 times that of the base. It shows that twins occupy a large part of the deformation at the state of ultra-low temperature, and twins are more likely to be produced at lower temperature than rolling at room temperature. This reflects that twinning is strongly influenced by temperature.

At low temperature, the twin critical shear stress of magnesium alloy is lower than that of pyramidal <a> and pyramidal <c + a> slips^17^. The {10$\overline{1}$2} <10$\overline{1}$1> of the twins has the smallest CRSS value, which is only greater than the basal <a> slip. According to the research, at room temperature, the CRSS value of basal slip of pure magnesium is 0.5–0.7 MPa, and has little relationship with temperature. The CRSS values of prismatic and pyramidal slips are as high as 40 MPa or more^18^, but it decreases significantly with increasing temperature. In Mg alloys {10$\overline{1}$1}, the CRSS required for compression twins is 76–153 MPa^19^, which is much greater than the CRSS (2–3 MPa) required for {10$\overline{1}$2} tensile twins^20,21^. In conclusion, the above reasons can also explain that 86° <11$\overline{2}$0> tensile twins are more likely to appear during ultra-low temperature rolling.

**Figure 7:**
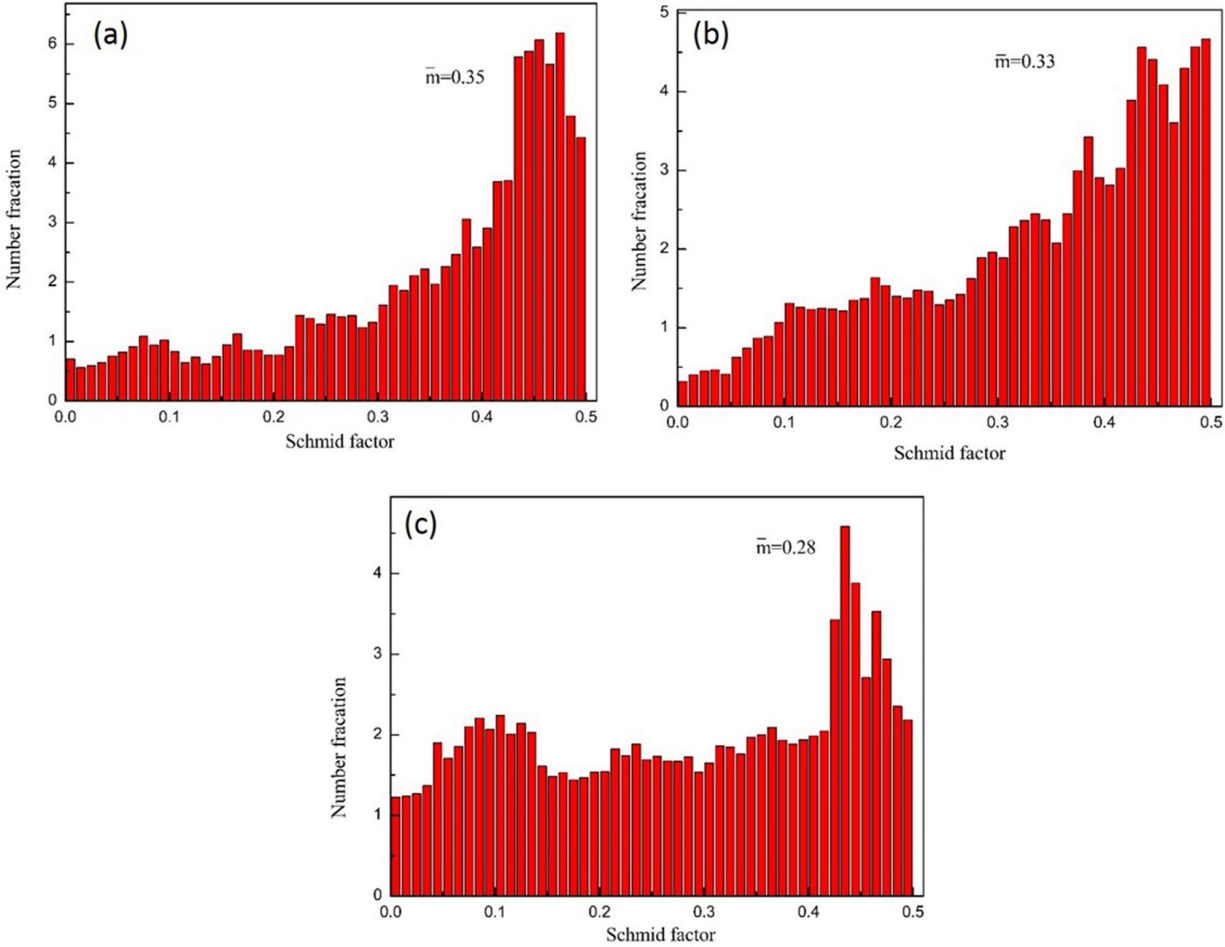
Schmid factor of Mg–2Y–0.6Nd–0.6Zr alloy at different rolling temperatures after ECAP. (**a**) ECAP4passes, (**b**) ECAP4Passes + rolling, (**c**) ECAP4Passes + liquid nitrogen.

Figure 7 shows the change of Schmid factor of rare earth magnesium alloy at different deformation temperatures after ECAP. As we all know, the larger the Schmid is, the easier the deformation will be. After 4 passes ECAP deformations, the average Schmid factor for basal slip is 0.35, which can provide favorable conditions for subsequent deformation, but after room temperature rolling and liquid nitrogen rolling, the Schmid factor decreases significantly, so that the basal slip is difficult to carry out. After rolling in liquid nitrogen, the increase of basal texture also reduces the slip deformation of magnesium alloy, which is dominated by twinning and grain rotation.

### Twin variants of magnesium alloys at low temperature

**Figure 8:**
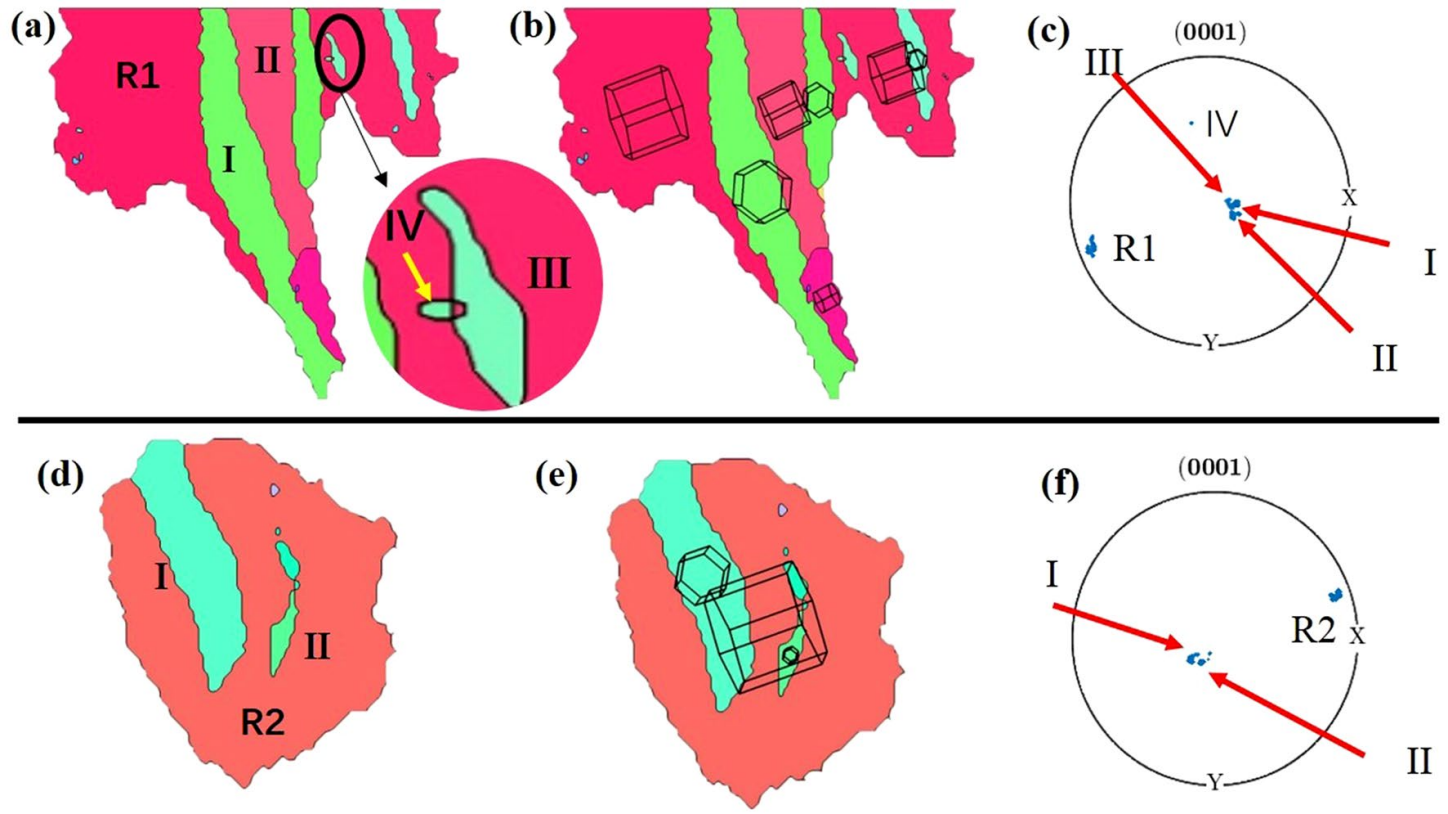
Selected grain regions R1, R2 in the Mg–2Y–0.6Nd–0.6Zr alloy rolled at room temperature. (**a**), (**d**) Inverse pole figure maps, (**b**), (**e**) three-dimensional stereoscopic orientation diagrams of individual grains, and their twins are shown in the (0001) pole figures in (**c**), (**f**).

Figure 8 shows the original grain regions R1, R2 selected in the room temperature rolled Mg–2Y–0.6Nd–0.6Zr alloy (Fig. 6a). Twins are marked in Fig. 8a and d, where {0001} pole figures of twins are shown in Fig. 8c and f, respectively, b and e are the orientation diagrams of grains R1 and R2. There are five twins in the crystal R1, four of which have the same twin variant and are parallel to each other and nucleate at different positions in the grain. In Fig. 8c, it can be found that twins “I”, “II” and “III” also have very similar orientations in the pole diagram. Of course, it can be observed from Fig. 8b that their 3D orientation diagrams are extremely similar, only rotated a little. However, the twin “IV” is different from the previous one, belonging to another twin variant of {10$\overline{1}$2} stretching twin. In grain R2, the number of twins is 2, but it is observed from Fig. 8d that the two twins tend to be parallel. Combined with Fig. 8e and f, it is also observed that their orientations are almost identical, so there is only one twin variant in grain R2.

**Figure 9:**
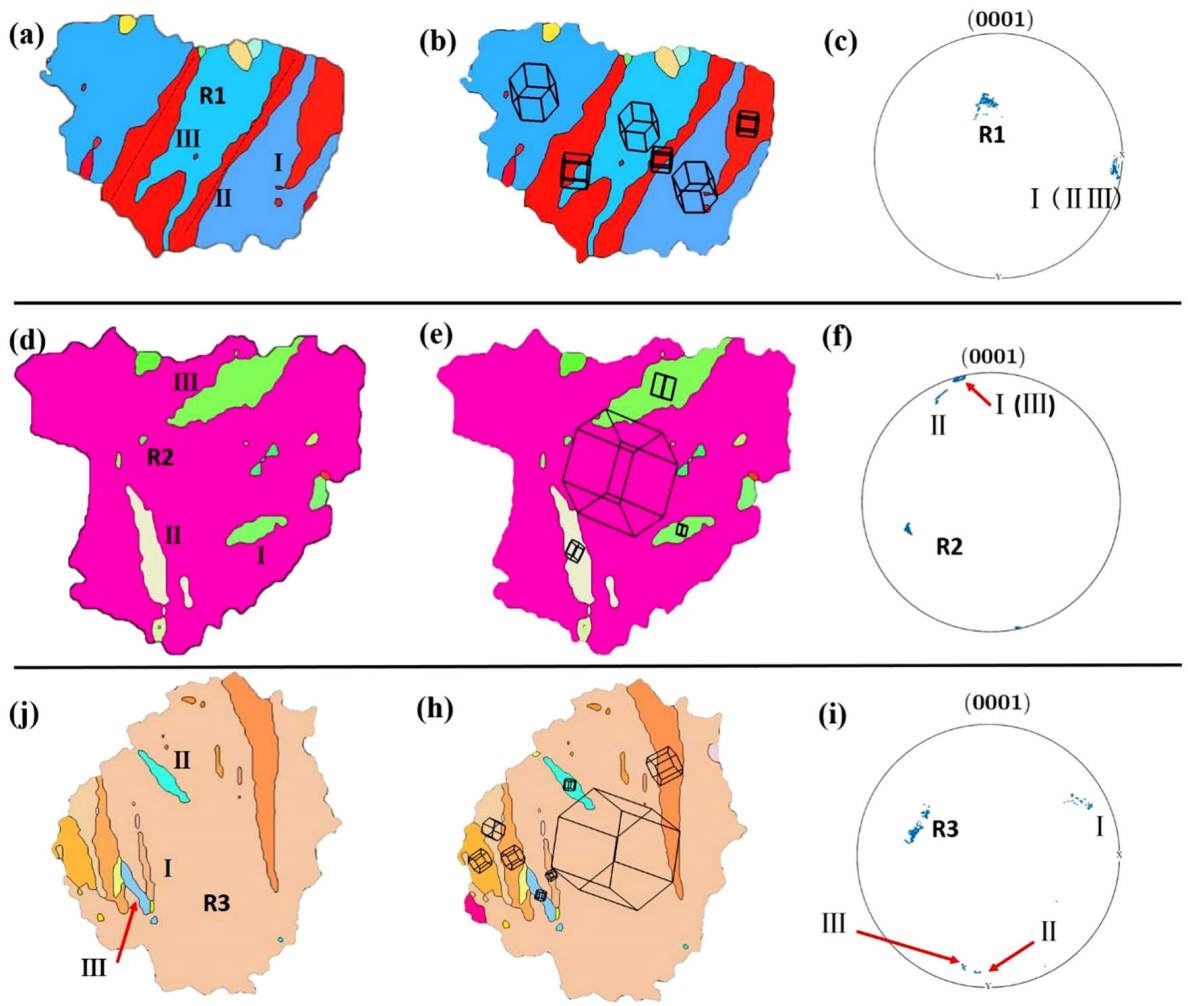
Selected grain area in ultra-low temperature rolling deformation Mg–2Y–0.6Nd–0.6Zr alloy. (**a**), (**d**), (**j**) Inverse pole figure maps, (**b**), (**e**), (**h**) three-dimensional stereoscopic orientation diagrams of individual grains, and their twins are shown in the (0001) pole figures in (**c**), (**f**), (**i**).

Figure 9 shows the original grain regions R1, R2 and R3 selected in the ultra-low temperature rolling deformation Mg–2Y–0.6Nd–0.6Zr alloy (Fig. 6b), with various variations of {10$\overline{1}$2}The effect of stretched twins on the evolution of crystal orientation: Fig. 9 shows twins and twin variants in different grains. Combined with Fig. 6c and d, it is observed that there are many {10$\overline{1}$2} <10$\overline{1}$1> stretching twins in grain R1 during ultra-low temperature rolling deformation, which have occupied the majority of the grain. Figure 9b show that the twin “I”, “II” and “III” are almost identical in crystal orientation. In addition, there are two twin variants in grain R2. Twins “II” and “III” are independent of each other in grain R2. In the grain R3, the twins “I” and “II” are cut, and the angle between the two is about 15°. At least 3 twins exist in the grain. Through observation and analysis of Fig. 9h and i, it is found that the twins “I”, “II”, and “III” all have different orientations, but the orientations of the twins “II” and “III” are not much different. The difference in the three-dimensional schematic diagram is also small. The twin “I” is different from the other two twins. Not only is the distribution on the (0001) pole figure very different from the others, but the difference can also be observed on the three-dimensional stereogram. In short, it can be obtained that there are at least two variants of {10$\overline{1}$2} tensile twins in grain R3.

**Figure 10:**
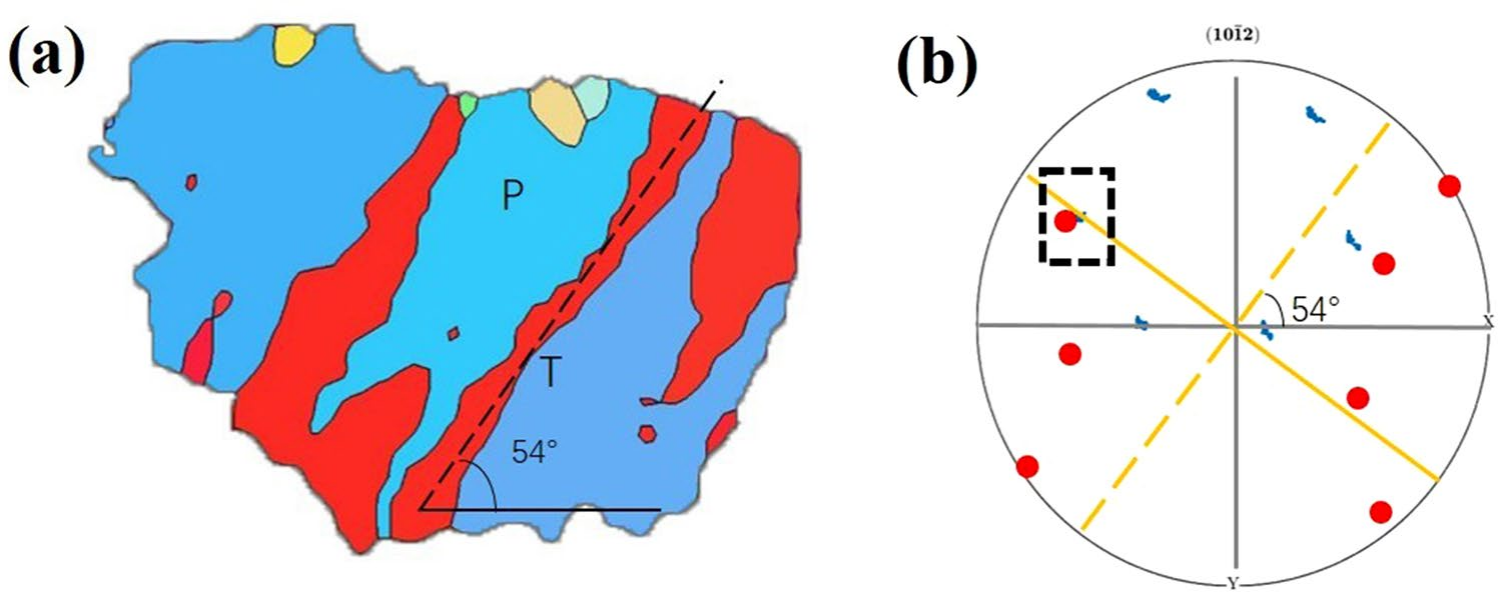
Inverse pole figure of the local EBSD twins of the specimen after ultra-low temperature rolling (**a**); {10 $\overline{1}$ 2} pole figures of the parent grain (P) and twin (T) (**b**).

Figure 10 shows the variant activated by ultra-low temperature rolling deformation. In Fig. 10a, the grains are selected in the local area of Fig. 6c. In this grain, a {10$\overline{1}$2} twin t occurs in the parent grain P during deformation, and the twin boundary of twin variant t is about 54° to the horizontal direction. Project P and T on the same {10$\overline{1}$2} pole figure, as shown in Fig. 10b, where the yellow dotted line represents the trace of the twin plane T. P and T are mirror-symmetrical about the {10$\overline{1}$2} plane and share this plane, so in theory the parent and twin have a {10$\overline{1}$2} plane pole that coincides, as shown in the figure. The pole inside the black box. The trace (yellow dotted line) of this point on the pole figure represents the intersection of the coincident variant and the observation plane. The angle between it and the horizontal direction is 54°, which is very close to the twin boundary. When observing other poles, most of their traces are inconsistent with the direction of the twin boundaries, and the above indicates that the variant corresponding to the parent P pole in the black frame is the actual twin variant.

### Analysis of mechanical properties

The corresponding tensile properties are shown in Table 1. It can be observed in the figure that after rolling at different temperatures, the strength and plasticity of the sample have changed greatly. After 4 passes of equal channel corner extrusion, the tensile strength of the material is 181 MPa, the yielding strength is 93.67 MPa and the elongation is 28.5%. After rolling, the properties of the materials have been improved to varying degrees. The tensile strength of the sample rolled at room temperature is 218.04 MPa and the yield strength is 174.79 MPa. The tensile strength of the sample deformed by ultra-low temperature rolling has reached 245.89 MPa, which is about 12.7% higher than that at room temperature and the yield strength is 216.21 MPa, an increase of about 23.69% compared to room temperature rolling. The main reason is that at low temperature and room temperature, Mg–2Y–0.6Nd–0.6Zr alloy has a large number of small grains formed inside the grains. At the same time, due to the work hardening, texture strengthening and twinning, the material is in during room temperature tensile deformation, twins hinder the plastic deformation behavior, so that the plasticity of the material is reduced, but the strength is improved^22^.

**Table 1:** Room temperature tensile properties of Mg–2Y–0.6Nd–0.6Zr alloy after ECAP at different rolling temperatures.

Sample	Ultimate tensile strength/MPa	Yield strength/MPa	Elongation %
ECAP 4passes	181	93.67	28.5
Room temperature	218.04	174.79	16
Liquid nitrogen temperature	245.89	216.21	11

Mg–2Y–0.6Nd–0.6Zr alloy has a strong basal texture after cold rolling and ultra-low temperature rolling. It can be seen from Fig. 4 that the basal surface texture strength is 9.38 and 10.78, respectively. Stretching along its ED (RD) direction, in polycrystalline materials, the relationship between yield strength and critical shear stress is:

1 $$\sigma = \frac{{\tau_{CRSS} }}{{\cos \varphi *\cos\uplambda }}$$

where σ is the yield strength, τCRSS is the critical shear stress, and m = cosφ * cosλ represents the orientation factor, also known as the Schmidt factor. The orientation factor has a great influence on the tensile yield strength of magnesium alloy materials. Earlier, EBSD was used to calculate the Schmid factor of the micro-basal slip. The Schmid factor of the basal slip decreases with the decrease of the deformation temperature. After rolling at room temperature and ultra-low temperature rolling, the Schmid factor is relatively small as 0.33 and 0.28, respectively. The larger the Schmid factor, the easier the basal slip is activated, but the opposite is not easy. When deformed at room temperature, the slip system is mainly based on the basal slip. For Mg–2Y–0.6Nd–0.6Zr alloy samples with a strong base texture, it will cause the phenomenon that the yield strength increases and the plasticity decreases in the tensile test, which is also the effect of texture strengthening^6^. Therefore, the tensile strength of ultra-low temperature rolling increased to 245.89 MPa, and the plasticity decreased compared with the four-pass deformation of equal channel angular extrusion and room temperature rolling deformation.

## Conclusions

1. The proportion of twins in room temperature rolling is 2.45%, and that in ultra-low temperature rolling is 4.23%. At the same amount of deformation, the lower the temperature, the more twins can be excited. Compared with cold rolling, there are more twins in liquid nitrogen rolling. At the same time, there are more variants of {10$\overline{1}$2} stretching twins during ultra-low temperature deformation, and the appearance of variants is mainly related to the Schmid factor.
2. After four passes of equal channel angular pressing, the texture of (0001) plane is 45° to the extrusion direction, and the texture gradually changed after ultra-low temperature rolling, thus forming a typical basal texture after ultra-low temperature rolling, and its maximum pole density is 10.78. After four passes of equal channel angular pressing, the Schmid factor is 0.35. After room temperature rolling and ultra-low temperature rolling, the Schmid factor is relatively small, which is 0.33 and 0.28 respectively, and the base slip is not easy to start.
3. Comparing the tensile properties of room temperature rolled samples and ultra-low temperature rolled samples, their yield strengths were 174.79 MPa and 216.21 MPa, respectively, and ultra-low temperature rolling increased about 23.69% compared to room temperature rolling. However, the rolled samples showed increased strength and decreased plasticity. This is the result of the combined effects of work hardening, texture strengthening and changes in twin content.
